# Supplementary material for: Vaccination coverage estimation in Mexico in children under five years old: Trends and associated factors
Source: PLoS One. 2021 Apr 16;16(4):e0250172. doi: 10.1371/journal.pone.0250172 (PMC8051786; doi:10.1371/journal.pone.0250172)
Supplement: S2 Table — BCG, Bacillus Calmette-Guerin vaccine; PV, complete cells pentavalent vaccine; PVa, acellular pentavalent vaccine; HB, Vaccine against Hepatitis B; Pnm, Conjugate vaccine against Pneumococcal; RV, Vaccine against rotavirus; MMR, Vaccine against measles, mumps, and rubella. (DOCX) [file pone.0250172.s002.docx]

**S2 Table. Estimated vaccination coverage of “With vaccination card” and “Without vaccination card” groups by age groups and survey year, Mexico 2000-2018.**

|  | **With vaccination card** | | | | |  | **Without vaccination card** | | | | |
| --- | --- | --- | --- | --- | --- | --- | --- | --- | --- | --- | --- |
|  | **Vaccine** | **Year** | **Coverage** | **95% Confidence Interval** | |  | **Vaccine** | **Year** | **Coverage** | **95% Confidence Interval** | |
|  |  |  |  | **Lower** | **Upper** |  |  |  |  | **Lower** | **Upper** |
| **Under 2 months** | |  |  |  |  |  |  |  |  |  |  |
|  | BCG | 2000 | 96.60 | 93.55 | 98.23 |  | - | - | - | - | - |
|  | BCG | 2006 | 89.48 | 76.10 | 95.78 |  | BCG | 2006 | 53.77 | 43.66 | 63.58 |
|  | BCG | 2010 | 98.18 | 94.50 | 99.41 |  | - | - | - | - | - |
|  | BCG | 2012 | 92.10 | 86.00 | 95.67 |  | BCG | 2012 | 18.29 | 11.52 | 27.79 |
|  | BCG | 2018 | 88.63 | 78.78 | 94.24 |  | BCG | 2018 | 78.91 | 68.72 | 86.44 |
|  | 1° dose HB | 2010 | 90.83 | 82.82 | 95.31 |  | HB | 2010 | 58.99 | 42.54 | 73.65 |
|  | 1° dose HB | 2012 | 87.50 | 80.29 | 92.33 |  | HB | 2012 | 45.16 | 35.59 | 55.10 |
|  | 1° dose HB | 2018 | 64.34 | 53.37 | 74.00 |  | HB | 2018 | 54.75 | 43.52 | 65.52 |
| **2 - 3 months** | |  |  |  |  |  |  |  |  |  |  |
|  | BCG | 2000 | 97.46 | 94.80 | 98.78 |  | - | - | - | - | - |
|  | BCG | 2006 | 92.90 | 87.20 | 96.17 |  | BCG | 2006 | 70.01 | 62.63 | 76.49 |
|  | BCG | 2010 | 98.82 | 94.96 | 99.73 |  | - | - | - | - | - |
|  | BCG | 2012 | 92.04 | 86.65 | 95.37 |  | BCG | 2012 | 18.75 | 11.00 | 30.11 |
|  | BCG | 2018 | 85.25 | 76.14 | 91.28 |  | BCG | 2018 | 89.55 | 81.44 | 94.37 |
|  | 1° dose HB | 2010 | 89.42 | 80.07 | 94.68 |  | HB | 2010 | 97.68 | 90.86 | 99.44 |
|  | 1° dose HB | 2012 | 88.79 | 81.48 | 93.45 |  | HB | 2012 | 55.79 | 42.13 | 68.63 |
|  | 1° dose HB | 2018 | 72.01 | 61.66 | 80.46 |  | HB | 2018 | 79.53 | 64.43 | 89.28 |
|  | 2° dose HB | 2010 | 69.49 | 58.78 | 78.44 |  | - | - | - | - | - |
|  | 2° dose HB | 2012 | 66.82 | 58.89 | 73.89 |  | - | - | - | - | - |
|  | 2° dose HB | 2018 | 20.09 | 12.42 | 30.82 |  | - | - | - | - | - |
|  | 1° dose PV | 2000 | 69.53 | 62.01 | 76.14 |  | - | - | - | - | - |
|  | 1° dose PV | 2006 | 73.99 | 66.25 | 80.47 |  | PV | 2006 | 69.36 | 46.30 | 85.60 |
|  | 1° dose Sabin | 2000 | 89.95 | 83.66 | 93.99 |  | - | - | - | - | - |
|  | 1° dose Sabin | 2006 | 78.36 | 71.46 | 83.97 |  | Sabin | 2006 | 81.93 | 58.26 | 93.65 |
|  | 1° dose PVa | 2010 | 74.10 | 63.45 | 82.50 |  | PVa | 2010 | 71.86 | 49.40 | 86.98 |
|  | 1° dose PVa | 2012 | 73.95 | 66.52 | 80.22 |  | PVa | 2012 | 57.75 | 43.78 | 70.59 |
|  | 1° dose PVa | 2018 | 66.36 | 55.35 | 75.85 |  | PVa | 2018 | 63.98 | 50.74 | 75.39 |
|  | 1° dose Pnm | 2010 | 64.94 | 54.49 | 74.13 |  | Pnm | 2010 | 41.54 | 24.62 | 60.72 |
|  | 1° dose Pnm | 2012 | 76.73 | 69.03 | 82.98 |  | Pnm | 2012 | 54.19 | 40.77 | 67.02 |
|  | 1° dose Pnm | 2018 | 76.28 | 66.06 | 84.16 |  | Pnm | 2018 | 62.54 | 49.49 | 73.98 |
|  | 1° dose RV | 2010 | 70.67 | 60.22 | 79.31 |  | RV | 2010 | 51.61 | 33.18 | 69.61 |
|  | 1° dose RV | 2012 | 66.79 | 58.75 | 73.96 |  | RV | 2012 | 51.12 | 38.17 | 63.93 |
|  | 1° dose RV | 2018 | 80.86 | 72.02 | 87.39 |  | RV | 2018 | 61.69 | 48.60 | 73.27 |
| **4 - 5 months** | |  |  |  |  |  |  |  |  |  |  |
|  | BCG | 2000 | 99.05 | 97.27 | 99.67 |  | - | - | - | - | - |
|  | BCG | 2006 | 94.12 | 88.91 | 96.96 |  | BCG | 2006 | 62.50 | 55.17 | 69.30 |
|  | BCG | 2010 | 99.82 | 99.21 | 99.96 |  | - | - | - | - | - |
|  | BCG | 2012 | 97.03 | 93.67 | 98.63 |  | BCG | 2012 | 8.00 | 3.74 | 16.28 |
|  | BCG | 2018 | 85.83 | 76.98 | 91.64 |  | BCG | 2018 | 94.82 | 88.68 | 97.72 |
|  | 1° dose HB | 2010 | 92.05 | 85.88 | 95.66 |  | HB | 2010 | 98.77 | 94.98 | 99.71 |
|  | 1° dose HB | 2012 | 96.48 | 93.09 | 98.24 |  | HB | 2012 | 62.53 | 49.36 | 74.07 |
|  | 1° dose HB | 2018 | 73.46 | 63.02 | 81.80 |  | HB | 2018 | 90.09 | 82.39 | 94.64 |
|  | 2° dose HB | 2010 | 96.37 | 92.70 | 98.23 |  | - | - | - | - | - |
|  | 2° dose HB | 2012 | 85.99 | 77.06 | 91.81 |  | - | - | - | - | - |
|  | 2° dose HB | 2018 | 37.74 | 25.56 | 51.68 |  | - | - | - | - | - |
|  | 1° dose PV | 2000 | 90.20 | 84.29 | 94.05 |  | - | - | - | - | - |
|  | 1° dose PV | 2006 | 83.12 | 76.58 | 88.12 |  | PV | 2006 | 82.88 | 64.81 | 92.71 |
|  | 2° dose PV | 2000 | 86.08 | 78.13 | 91.45 |  | - | - | - | - | - |
|  | 2° dose PV | 2006 | 67.09 | 59.59 | 73.82 |  | - | - | - | - | - |
|  | 1° dose Sabin | 2000 | 99.20 | 97.71 | 99.73 |  | Sabin | 2000 | 95.43 | 86.48 | 98.55 |
|  | 1° dose Sabin | 2006 | 92.49 | 86.72 | 95.88 |  | - | - | - | - | - |
|  | 2° dose Sabin | 2000 | 98.98 | 97.08 | 99.65 |  | - | - | - | - | - |
|  | 2° dose Sabin | 2006 | 71.12 | 63.79 | 77.50 |  | - | - | - | - | - |
|  | 1° dose PVa | 2010 | 94.19 | 90.21 | 96.62 |  | PVa | 2010 | 97.19 | 92.82 | 98.93 |
|  | 1° dose PVa | 2012 | 89.48 | 80.41 | 94.63 |  | PVa | 2012 | 77.60 | 65.80 | 86.18 |
|  | 1° dose PVa | 2018 | 84.33 | 75.52 | 90.38 |  | PVa | 2018 | 82.63 | 72.37 | 89.63 |
|  | 2° dose PVa | 2010 | 71.48 | 63.67 | 78.18 |  | - | - | - | - | - |
|  | 2° dose PVa | 2012 | 64.72 | 55.89 | 72.65 |  | - | - | - | - | - |
|  | 2° dose PVa | 2018 | 47.53 | 35.57 | 59.77 |  | - | - | - | - | - |
|  | 1° dose Pnm | 2010 | 81.01 | 73.88 | 86.55 |  | Pnm | 2010 | 56.72 | 37.13 | 74.42 |
|  | 1° dose Pnm | 2012 | 93.35 | 83.21 | 97.54 |  | Pnm | 2012 | 71.98 | 58.69 | 82.29 |
|  | 1° dose Pnm | 2018 | 86.88 | 78.86 | 92.16 |  | Pnm | 2018 | 76.66 | 66.41 | 84.51 |
|  | 2° dose Pnm | 2010 | 52.77 | 44.53 | 60.87 |  | - | - | - | - | - |
|  | 2° dose Pnm | 2012 | 67.80 | 59.09 | 75.42 |  | - | - | - | - | - |
|  | 2° dose Pnm | 2018 | 49.44 | 37.24 | 61.70 |  | - | - | - | - | - |
|  | 1° dose RV | 2010 | 87.59 | 80.57 | 92.32 |  | RV | 2010 | 96.35 | 92.03 | 98.37 |
|  | 1° dose RV | 2012 | 77.08 | 66.41 | 85.12 |  | RV | 2012 | 75.31 | 63.39 | 84.30 |
|  | 1° dose RV | 2018 | 82.10 | 72.12 | 89.05 |  | RV | 2018 | 79.28 | 69.11 | 86.74 |
|  | 2° dose RV | 2010 | 58.99 | 50.72 | 66.78 |  | - | - | - | - | - |
|  | 2° dose RV | 2012 | 48.66 | 39.84 | 57.57 |  | - | - | - | - | - |
|  | 2° dose RV | 2018 | 59.10 | 45.73 | 71.24 |  | - | - | - | - | - |
| **6 - 11 months** | |  |  |  |  |  |  |  |  |  |  |
|  | BCG | 2000 | 97.59 | 95.43 | 98.74 |  | - | - | - | - | - |
|  | BCG | 2006 | 96.17 | 93.05 | 97.91 |  | BCG | 2006 | 66.22 | 60.93 | 71.13 |
|  | BCG | 2010 | 99.14 | 97.77 | 99.67 |  | - | - | - | - | - |
|  | BCG | 2012 | 95.73 | 92.97 | 97.44 |  | BCG | 2012 | 25.14 | 18.01 | 33.93 |
|  | BCG | 2018 | 90.01 | 86.26 | 92.82 |  | BCG | 2018 | 92.07 | 87.69 | 94.97 |
|  | 1° dose HB | 2010 | 90.29 | 86.54 | 93.08 |  | HB | 2010 | 96.91 | 89.26 | 99.16 |
|  | 1° dose HB | 2012 | 94.69 | 91.52 | 96.72 |  | HB | 2012 | 73.57 | 66.01 | 79.95 |
|  | 1° dose HB | 2018 | 78.49 | 72.85 | 83.23 |  | HB | 2018 | 86.26 | 80.74 | 90.39 |
|  | 2° dose HB | 2010 | 95.75 | 93.71 | 97.16 |  | - | - | - | - | - |
|  | 2° dose HB | 2012 | 95.37 | 92.62 | 97.13 |  | - | - | - | - | - |
|  | 2° dose HB | 2018 | 36.74 | 31.14 | 42.71 |  | - | - | - | - | - |
|  | 3° dose HB | 2010 | 72.07 | 66.99 | 76.63 |  | - | - | - | - | - |
|  | 3° dose HB | 2012 | 80.73 | 76.37 | 84.45 |  | - | - | - | - | - |
|  | 3° dose HB | 2018 | 22.23 | 17.78 | 27.42 |  | - | - | - | - | - |
|  | 1° dose PV | 2000 | 93.61 | 91.45 | 95.26 |  | - | - | - | - | - |
|  | 1° dose PV | 2006 | 90.34 | 86.57 | 93.14 |  | PV | 2006 | 80.95 | 56.31 | 93.34 |
|  | 2° dose PV | 2000 | 93.20 | 90.91 | 94.95 |  | - | - | - | - | - |
|  | 2° dose PV | 2006 | 87.56 | 83.52 | 90.71 |  | - | - | - | - | - |
|  | 3° dose PV | 2000 | 91.78 | 89.03 | 93.89 |  | - | - | - | - | - |
|  | 3° dose PV | 2006 | 73.23 | 68.37 | 77.58 |  | - | - | - | - | - |
|  | 1° dose Sabin | 2000 | 98.81 | 96.82 | 99.56 |  | - | - | - | - | - |
|  | 1° dose Sabin | 2006 | 96.06 | 92.69 | 97.91 |  | Sabin | 2006 | 93.16 | 84.25 | 97.20 |
|  | 2° dose Sabin | 2000 | 98.76 | 96.68 | 99.54 |  | - | - | - | - | - |
|  | 2° dose Sabin | 2006 | 93.60 | 90.14 | 95.90 |  | - | - | - | - | - |
|  | 3° dose Sabin | 2000 | 98.53 | 96.08 | 99.46 |  | - | - | - | - | - |
|  | 3° dose Sabin | 2006 | 80.82 | 76.27 | 84.67 |  | - | - | - | - | - |
|  | 1° dose PVa | 2010 | 97.74 | 94.32 | 99.12 |  | PVa | 2010 | 95.06 | 88.16 | 98.03 |
|  | 1° dose PVa | 2012 | 96.66 | 94.23 | 98.09 |  | PVa | 2012 | 86.24 | 78.72 | 91.39 |
|  | 1° dose PVa | 2018 | 90.09 | 86.24 | 92.95 |  | PVa | 2018 | 83.55 | 77.29 | 88.35 |
|  | 2° dose PVa | 2010 | 87.92 | 83.26 | 91.41 |  | - | - | - | - | - |
|  | 2° dose PVa | 2012 | 88.67 | 85.42 | 91.28 |  | - | - | - | - | - |
|  | 2° dose PVa | 2018 | 80.71 | 75.59 | 84.98 |  | - | - | - | - | - |
|  | 3° dose PVa | 2010 | 69.63 | 64.53 | 74.28 |  | - | - | - | - | - |
|  | 3° dose PVa | 2012 | 68.39 | 63.61 | 72.81 |  | - | - | - | - | - |
|  | 3° dose PVa | 2018 | 62.34 | 56.11 | 68.20 |  | - | - | - | - | - |
|  | 1° dose Pnm | 2010 | 88.74 | 85.19 | 91.52 |  | Pnm | 2010 | 91.85 | 85.06 | 95.71 |
|  | 1° dose Pnm | 2012 | 95.50 | 93.03 | 97.12 |  | Pnm | 2012 | 78.04 | 69.93 | 84.45 |
|  | 1° dose Pnm | 2018 | 90.76 | 87.14 | 93.43 |  | Pnm | 2018 | 79.78 | 73.14 | 85.11 |
|  | 2° dose Pnm | 2010 | 73.85 | 68.93 | 78.23 |  | - | - | - | - | - |
|  | 2° dose Pnm | 2012 | 88.52 | 85.08 | 91.25 |  | - | - | - | - | - |
|  | 2° dose Pnm | 2018 | 81.27 | 76.13 | 85.51 |  | - | - | - | - | - |
|  | 1° dose RV | 2010 | 92.39 | 89.26 | 94.67 |  | RV | 2010 | 93.93 | 87.06 | 97.27 |
|  | 1° dose RV | 2012 | 87.31 | 83.72 | 90.21 |  | RV | 2012 | 80.82 | 73.25 | 86.65 |
|  | 1° dose RV | 2018 | 90.62 | 86.92 | 93.35 |  | RV | 2018 | 80.57 | 73.57 | 86.07 |
|  | 2° dose RV | 2010 | 78.10 | 73.48 | 82.11 |  | - | - | - | - | - |
|  | 2° dose RV | 2012 | 68.89 | 63.95 | 73.43 |  | - | - | - | - | - |
|  | 2° dose RV | 2018 | 83.16 | 78.30 | 87.11 |  | - | - | - | - | - |
|  | 3° dose RV | 2018 | 66.45 | 60.15 | 72.22 |  | - | - | - | - | - |
| **12 - 23 months** | |  |  |  |  |  |  |  |  |  |  |
|  | BCG | 2000 | 97.74 | 95.26 | 98.93 |  | - | - | - | - | - |
|  | BCG | 2006 | 96.99 | 95.27 | 98.09 |  | BCG | 2006 | 73.58 | 70.55 | 76.40 |
|  | BCG | 2010 | 99.04 | 98.36 | 99.45 |  | - | - | - | - | - |
|  | BCG | 2012 | 96.59 | 95.19 | 97.59 |  | BCG | 2012 | 18.19 | 14.22 | 22.97 |
|  | BCG | 2018 | 96.31 | 94.65 | 97.48 |  | BCG | 2018 | 94.00 | 91.65 | 95.72 |
|  | 1° dose HB | 2010 | 91.30 | 88.71 | 93.34 |  | HB | 2010 | 98.20 | 96.40 | 99.11 |
|  | 1° dose HB | 2012 | 96.68 | 95.14 | 97.74 |  | HB | 2012 | 74.77 | 70.30 | 78.78 |
|  | 1° dose HB | 2018 | 90.32 | 87.21 | 92.74 |  | HB | 2018 | 92.55 | 89.80 | 94.61 |
|  | 2° dose HB | 2010 | 94.73 | 92.77 | 96.19 |  | - | - | - | - | - |
|  | 2° dose HB | 2012 | 97.15 | 95.84 | 98.06 |  | - | - | - | - | - |
|  | 2° dose HB | 2018 | 57.36 | 53.09 | 61.52 |  | - | - | - | - | - |
|  | 3° dose HB | 2010 | 91.40 | 89.11 | 93.24 |  | - | - | - | - | - |
|  | 3° dose HB | 2012 | 92.18 | 89.92 | 93.96 |  | - | - | - | - | - |
|  | 3° dose HB | 2018 | 50.72 | 46.48 | 54.95 |  | - | - | - | - | - |
|  | 1° dose PV | 2000 | 98.30 | 97.10 | 99.01 |  | PV | 2000 | 90.93 | 79.91 | 96.19 |
|  | 1° dose PV | 2006 | 97.72 | 96.58 | 98.49 |  | - | - | - | - | - |
|  | 2° dose PV | 2000 | 98.26 | 97.04 | 98.99 |  | - | - | - | - | - |
|  | 2° dose PV | 2006 | 97.47 | 96.32 | 98.26 |  | - | - | - | - | - |
|  | 3° dose PV | 2000 | 98.18 | 96.90 | 98.94 |  | - | - | - | - | - |
|  | 3° dose PV | 2006 | 93.06 | 90.63 | 94.90 |  | - | - | - | - | - |
|  | 1° dose Sabin | 2000 | 99.29 | 97.91 | 99.76 |  | - | - | - | - | - |
|  | 1° dose Sabin | 2006 | 98.47 | 97.38 | 99.11 |  | Sabin | 2006 | 97.54 | 94.24 | 98.97 |
|  | 2° dose Sabin | 2000 | 99.27 | 97.87 | 99.75 |  | - | - | - | - | - |
|  | 2° dose Sabin | 2006 | 98.07 | 96.97 | 98.78 |  | - | - | - | - | - |
|  | 3° dose Sabin | 2000 | 99.25 | 97.81 | 99.75 |  | - | - | - | - | - |
|  | 3° dose Sabin | 2006 | 96.18 | 94.36 | 97.43 |  | - | - | - | - | - |
|  | MMR | 2000 | 74.11 | 70.89 | 77.09 |  | - | - | - | - | - |
|  | MMR | 2006 | 78.86 | 75.70 | 81.70 |  | MMR | 2006 | 81.21 | 70.13 | 88.84 |
|  | MMR | 2010 | 72.48 | 68.56 | 76.07 |  | MMR | 2010 | 89.49 | 85.20 | 92.64 |
|  | MMR | 2012 | 79.85 | 76.71 | 82.66 |  | MMR | 2012 | 72.91 | 68.25 | 77.11 |
|  | MMR | 2018 | 63.07 | 58.77 | 67.18 |  | MMR | 2018 | 57.71 | 53.23 | 62.07 |
|  | 1° dose PVa | 2010 | 98.50 | 97.32 | 99.16 |  | PVa | 2010 | 97.73 | 95.75 | 98.80 |
|  | 1° dose PVa | 2012 | 97.75 | 95.76 | 98.81 |  | PVa | 2012 | 88.33 | 84.41 | 91.36 |
|  | 1° dose PVa | 2018 | 94.99 | 92.65 | 96.62 |  | PVa | 2018 | 90.03 | 87.38 | 92.17 |
|  | 2° dose PVa | 2010 | 97.12 | 95.13 | 98.31 |  | - | - | - | - | - |
|  | 2° dose PVa | 2012 | 96.61 | 94.78 | 97.81 |  | - | - | - | - | - |
|  | 2° dose PVa | 2018 | 91.47 | 88.47 | 93.75 |  | - | - | - | - | - |
|  | 3° dose PVa | 2010 | 91.05 | 88.20 | 93.26 |  | - | - | - | - | - |
|  | 3° dose PVa | 2012 | 90.84 | 88.44 | 92.79 |  | - | - | - | - | - |
|  | 3° dose PVa | 2018 | 84.52 | 81.10 | 87.41 |  | - | - | - | - | - |
|  | 1° dose Pnm | 2010 | 88.96 | 86.50 | 91.02 |  | Pnm | 2010 | 93.08 | 89.73 | 95.39 |
|  | 1° dose Pnm | 2012 | 95.10 | 93.26 | 96.46 |  | Pnm | 2012 | 85.60 | 81.99 | 88.58 |
|  | 1° dose Pnm | 2018 | 94.52 | 92.13 | 96.21 |  | Pnm | 2018 | 87.15 | 84.01 | 89.74 |
|  | 2° dose Pnm | 2010 | 78.95 | 75.36 | 82.14 |  | - | - | - | - | - |
|  | 2° dose Pnm | 2012 | 91.47 | 88.95 | 93.46 |  | - | - | - | - | - |
|  | 2° dose Pnm | 2018 | 91.59 | 88.77 | 93.75 |  | - | - | - | - | - |
|  | 3° dose Pnm | 2018 | 68.87 | 64.65 | 72.79 |  | - | - | - | - | - |
|  | 1° dose RV | 2010 | 88.72 | 85.62 | 91.22 |  | RV | 2010 | 93.90 | 90.85 | 95.98 |
|  | 1° dose RV | 2012 | 90.72 | 88.41 | 92.61 |  | RV | 2012 | 83.13 | 78.52 | 86.91 |
|  | 1° dose RV | 2018 | 94.05 | 91.56 | 95.84 |  | RV | 2018 | 89.54 | 86.68 | 91.84 |
|  | 2° dose RV | 2010 | 81.44 | 78.14 | 84.35 |  | - | - | - | - | - |
|  | 2° dose RV | 2012 | 80.41 | 77.15 | 83.31 |  | - | - | - | - | - |
|  | 2° dose RV | 2018 | 87.36 | 84.13 | 90.01 |  | - | - | - | - | - |
|  | 3° dose RV | 2018 | 68.47 | 64.34 | 72.33 |  | - | - | - | - | - |
| **24 - 35 months** | |  |  |  |  |  |  |  |  |  |  |
|  | BCG | 2000 | 98.65 | 97.37 | 99.32 |  | - | - | - | - | - |
|  | BCG | 2006 | 97.37 | 95.68 | 98.41 |  | BCG | 2006 | 70.64 | 67.46 | 73.64 |
|  | BCG | 2010 | 98.73 | 97.59 | 99.33 |  | BCG | 2010 | 98.53 | 96.32 | 99.42 |
|  | BCG | 2012 | 96.54 | 94.40 | 97.88 |  | BCG | 2012 | 16.55 | 13.29 | 20.42 |
|  | BCG | 2018 | 98.42 | 97.33 | 99.07 |  | BCG | 2018 | 94.23 | 91.89 | 95.92 |
|  | 1° dose HB | 2012 | 95.33 | 93.14 | 96.85 |  | - | - | - | - | - |
|  | 1° dose HB | 2018 | 98.43 | 96.98 | 99.19 |  | HB | 2018 | 77.62 | 73.88 | 80.96 |
|  | 1° dose HB | 2010 | 91.30 | 88.71 | 93.34 |  | HB | 2010 | 94.10 | 91.63 | 95.87 |
|  | 2° dose HB | 2010 | 94.73 | 92.77 | 96.19 |  | - | - | - | - | - |
|  | 2° dose HB | 2012 | 97.11 | 95.56 | 98.12 |  | - | - | - | - | - |
|  | 2° dose HB | 2018 | 75.41 | 71.83 | 78.67 |  | - | - | - | - | - |
|  | 3° dose HB | 2010 | 91.40 | 89.11 | 93.24 |  | - | - | - | - | - |
|  | 3° dose HB | 2012 | 95.86 | 94.11 | 97.10 |  | - | - | - | - | - |
|  | 3° dose HB | 2018 | 84.11 | 80.91 | 86.87 |  | - | - | - | - | - |
|  | 1° dose PV | 2000 | 99.49 | 98.84 | 99.78 |  | PV | 2000 | 94.16 | 87.59 | 97.36 |
|  | 1° dose PV | 2006 | 98.44 | 97.41 | 99.06 |  | - | - | - | - | - |
|  | 2° dose PV | 2000 | 99.48 | 98.81 | 99.77 |  | - | - | - | - | - |
|  | 2° dose PV | 2006 | 97.46 | 96.10 | 98.35 |  | - | - | - | - | - |
|  | 3° dose PV | 2000 | 99.46 | 98.78 | 99.77 |  | - | - | - | - | - |
|  | 3° dose PV | 2006 | 97.20 | 95.75 | 98.16 |  | - | - | - | - | - |
|  | 1° dose Sabin | 2000 | 99.88 | 99.29 | 99.98 |  | - | - | - | - | - |
|  | 1° dose Sabin | 2006 | 98.73 | 97.71 | 99.30 |  | Sabin | 2006 | 98.82 | 96.62 | 99.60 |
|  | 2° dose Sabin | 2000 | 99.88 | 99.28 | 99.98 |  | - | - | - | - | - |
|  | 2° dose Sabin | 2006 | 97.99 | 96.61 | 98.81 |  | - | - | - | - | - |
|  | 3° dose Sabin | 2000 | 99.88 | 99.26 | 99.98 |  | - | - | - | - | - |
|  | 3° dose Sabin | 2006 | 97.66 | 96.30 | 98.53 |  | - | - | - | - | - |
|  | MMR | 2000 | 89.41 | 87.02 | 91.40 |  | - | - | - | - | - |
|  | MMR | 2006 | 93.50 | 91.48 | 95.07 |  | MMR | 2006 | 91.83 | 83.95 | 96.02 |
|  | MMR | 2010 | 83.23 | 80.29 | 85.82 |  | MMR | 2010 | 87.57 | 81.55 | 91.82 |
|  | MMR | 2012 | 88.27 | 85.40 | 90.63 |  | MMR | 2012 | 85.91 | 82.33 | 88.86 |
|  | MMR | 2018 | 85.93 | 82.96 | 88.45 |  | MMR | 2018 | 71.43 | 67.30 | 75.22 |
|  | 1° dose PVa | 2010 | 96.69 | 94.95 | 97.85 |  | - | - | - | - | - |
|  | 1° dose PVa | 2012 | 98.73 | 97.72 | 99.29 |  | PVa | 2012 | 91.01 | 87.69 | 93.50 |
|  | 1° dose PVa | 2018 | 97.97 | 96.66 | 98.77 |  | PVa | 2018 | 91.39 | 88.71 | 93.48 |
|  | 2° dose PVa | 2010 | 93.58 | 90.62 | 95.65 |  | - | - | - | - | - |
|  | 2° dose PVa | 2012 | 97.79 | 96.57 | 98.59 |  | - | - | - | - | - |
|  | 2° dose PVa | 2018 | 97.86 | 96.62 | 98.65 |  | - | - | - | - | - |
|  | 3° dose PVa | 2010 | 92.75 | 89.68 | 94.96 |  | - | - | - | - | - |
|  | 3° dose PVa | 2012 | 95.25 | 93.21 | 96.70 |  | - | - | - | - | - |
|  | 3° dose PVa | 2018 | 94.93 | 93.07 | 96.31 |  | - | - | - | - | - |
|  | 1° dose Pnm | 2010 | 88.96 | 86.50 | 91.02 |  | - | - | - | - | - |
|  | 1° dose Pnm | 2012 | 94.99 | 93.37 | 96.23 |  | Pnm | 2012 | 83.81 | 79.71 | 87.21 |
|  | 1° dose Pnm | 2018 | 98.53 | 97.45 | 99.16 |  | Pnm | 2018 | 89.65 | 86.78 | 91.95 |
|  | 2° dose Pnm | 2010 | 21.05 | 17.86 | 24.64 |  | - | - | - | - | - |
|  | 2° dose Pnm | 2012 | 91.07 | 88.83 | 92.89 |  | - | - | - | - | - |
|  | 2° dose Pnm | 2018 | 96.75 | 94.94 | 97.93 |  | - | - | - | - | - |
|  | 3° dose Pnm | 2018 | 86.71 | 83.73 | 89.21 |  | - | - | - | - | - |
|  | 1° dose RV | 2010 | 88.72 | 85.62 | 91.22 |  | - | - | - | - | - |
|  | 1° dose RV | 2012 | 94.10 | 92.35 | 95.46 |  | RV | 2012 | 88.10 | 84.15 | 91.17 |
|  | 1° dose RV | 2018 | 95.81 | 93.56 | 97.29 |  | RV | 2018 | 90.17 | 87.21 | 92.51 |
|  | 2° dose RV | 2010 | 81.44 | 78.14 | 84.35 |  | - | - | - | - | - |
|  | 2° dose RV | 2012 | 87.14 | 84.66 | 89.27 |  | - | - | - | - | - |
|  | 2° dose RV | 2018 | 87.80 | 84.71 | 90.34 |  | - | - | - | - | - |
|  | 3° dose RV | 2018 | 69.30 | 65.35 | 72.99 |  | - | - | - | - | - |
|  |  |  |  |  |  |  |  |  |  |  |  |
| BCG, Bacillus Calmette-Guerin vaccine; PV, complete cells pentavalent vaccine; PVa, acellular pentavalent vaccine; HB, Vaccine against Hepatitis B; Pnm, Conjugate vaccine against Pneumococcal; RV, Vaccine against rotavirus; MMR, Vaccine against measles, mumps, and rubella. | | | | | | | | | | | |
