# Supplementary material for: Vaccination coverage estimation in Mexico in children under five years old: Trends and associated factors
Source: PLoS One. 2021 Apr 16;16(4):e0250172. doi: 10.1371/journal.pone.0250172 (PMC8051786; doi:10.1371/journal.pone.0250172)
Supplement: Attachment — Submitted filename: Response to Reviewers.docx [file pone.0250172.s003.docx]

**Response to Journal Requirements and Reviewers**

Journal Requirements:

When submitting your revision, we need you to address these additional requirements.

1. Please ensure that your manuscript meets PLOS ONE's style requirements, including those for file naming. The PLOS ONE style templates can be found at

[https://journals.plos.org/plosone/s/file?id=wjVg/PLOSOne_formatting_sample_main_body.pdf](https://na01.safelinks.protection.outlook.com/?url=https%3A%2F%2Fjournals.plos.org%2Fplosone%2Fs%2Ffile%3Fid%3DwjVg%2FPLOSOne_formatting_sample_main_body.pdf&data=04%7C01%7C%7C2d969f51ff8740f942ad08d8c4903fb6%7C84df9e7fe9f640afb435aaaaaaaaaaaa%7C1%7C0%7C637475471122618717%7CUnknown%7CTWFpbGZsb3d8eyJWIjoiMC4wLjAwMDAiLCJQIjoiV2luMzIiLCJBTiI6Ik1haWwiLCJXVCI6Mn0%3D%7C1000&sdata=6hkDEUZsG4AcUN4ZaHcBvFzaCnJW14%2FkrgFd9FgTFwc%3D&reserved=0) and

[https://journals.plos.org/plosone/s/file?id=ba62/PLOSOne_formatting_sample_title_authors_affiliations.pdf](https://na01.safelinks.protection.outlook.com/?url=https%3A%2F%2Fjournals.plos.org%2Fplosone%2Fs%2Ffile%3Fid%3Dba62%2FPLOSOne_formatting_sample_title_authors_affiliations.pdf&data=04%7C01%7C%7C2d969f51ff8740f942ad08d8c4903fb6%7C84df9e7fe9f640afb435aaaaaaaaaaaa%7C1%7C0%7C637475471122618717%7CUnknown%7CTWFpbGZsb3d8eyJWIjoiMC4wLjAwMDAiLCJQIjoiV2luMzIiLCJBTiI6Ik1haWwiLCJXVCI6Mn0%3D%7C1000&sdata=sI9ZNZDE1dvxxuiTDHgrWPxv7tOpOiD85YgBa1E5qKM%3D&reserved=0)

**Response:** Thank you for the observation. We checked the submitted manuscript and found some PLOS ONE's style mistakes, we apologize for that, we did change in the main manuscript**.**

2.Please provide additional details regarding participant consent. In the ethics statement in the Methods and online submission information, please ensure that you have specified (1) whether consent was informed and (2) what type you obtained (for instance, written or verbal, and if verbal, how it was documented and witnessed). If your study included minors, state whether you obtained consent from parents or guardians. If the need for consent was waived by the ethics committee, please include this information.

If you are reporting a retrospective study of medical records or archived samples, please ensure that you have discussed whether all data were fully anonymized before you accessed them and/or whether the IRB or ethics committee waived the requirement for informed consent. If patients provided informed written consent to have data from their medical records used in research, please include this information.

**Response:**

We added the following paragraph in the data sources part of the Methodology:

“A secondary analysis from five Mexican national health surveys (2000 [23], 2006 [24], 2010 [25], 2012 [26], and 2018 [27]) was carried out, whose design was probabilistic with national and state representativeness. The surveys content and protocols were approved by the ethics and research committee at the National Institute of Public Health (INSP, by its acronym in Spanish) of Mexico. Data collection was carried out by both INSP and National Institute of Statistic and Geography (INEGI, by its acronym in Spanish), household questionnaires and individual questionnaires were used [23-27]. All participants in the surveys provided oral and/or written consents, where information was provided on the objectives of the research, the voluntary nature of participation and the confidentiality of the information. For children under five years old, mother or guardian (caretaker) were asked to provide information on the vaccination of their children. All the information collected was anonymized and published in digital repositories. More detail was published elsewhere [23-27].”

3.Please include your tables as part of your main manuscript and remove the individual files. Please note that supplementary tables (should remain/ be uploaded) as separate "supporting information" files.

**Response:** Sincere apologies for the error in the submitted version. We included them in the main revised manuscript**.**

4.We note that Figure(s) 2A and 2B in your submission contain map images which may be copyrighted. All PLOS content is published under the Creative Commons Attribution License (CC BY 4.0), which means that the manuscript, images, and Supporting Information files will be freely available online, and any third party is permitted to access, download, copy, distribute, and use these materials in any way, even commercially, with proper attribution. For these reasons, we cannot publish previously copyrighted maps or satellite images created using proprietary data, such as Google software (Google Maps, Street View, and Earth). For more information, see our copyright guidelines: [http://journals.plos.org/plosone/s/licenses-and-copyright](https://na01.safelinks.protection.outlook.com/?url=http%3A%2F%2Fjournals.plos.org%2Fplosone%2Fs%2Flicenses-and-copyright&data=04%7C01%7C%7C2d969f51ff8740f942ad08d8c4903fb6%7C84df9e7fe9f640afb435aaaaaaaaaaaa%7C1%7C0%7C637475471122628712%7CUnknown%7CTWFpbGZsb3d8eyJWIjoiMC4wLjAwMDAiLCJQIjoiV2luMzIiLCJBTiI6Ik1haWwiLCJXVCI6Mn0%3D%7C1000&sdata=pMFBR%2B8SjMnbRaiAd6wo7ojTv7ukba1SOfO438bHCRc%3D&reserved=0).

We require you to either (1) present written permission from the copyright holder to publish these figures specifically under the CC BY 4.0 license, or (2) remove the figures from your submission:

a) You may seek permission from the original copyright holder of Figure(s) 2A and 2B to publish the content specifically under the CC BY 4.0 license.

We recommend that you contact the original copyright holder with the Content Permission Form ([http://journals.plos.org/plosone/s/file?id=7c09/content-permission-form.pdf](https://na01.safelinks.protection.outlook.com/?url=http%3A%2F%2Fjournals.plos.org%2Fplosone%2Fs%2Ffile%3Fid%3D7c09%2Fcontent-permission-form.pdf&data=04%7C01%7C%7C2d969f51ff8740f942ad08d8c4903fb6%7C84df9e7fe9f640afb435aaaaaaaaaaaa%7C1%7C0%7C637475471122628712%7CUnknown%7CTWFpbGZsb3d8eyJWIjoiMC4wLjAwMDAiLCJQIjoiV2luMzIiLCJBTiI6Ik1haWwiLCJXVCI6Mn0%3D%7C1000&sdata=s%2FqyPzvTfP%2FlWj2nJW3Y8Lt7NYSzS4%2Fmr%2BqMYXWQtL0%3D&reserved=0)) and the following text:

“I request permission for the open-access journal PLOS ONE to publish XXX under the Creative Commons Attribution License (CCAL) CC BY 4.0 ([http://creativecommons.org/licenses/by/4.0/](https://na01.safelinks.protection.outlook.com/?url=http%3A%2F%2Fcreativecommons.org%2Flicenses%2Fby%2F4.0%2F&data=04%7C01%7C%7C2d969f51ff8740f942ad08d8c4903fb6%7C84df9e7fe9f640afb435aaaaaaaaaaaa%7C1%7C0%7C637475471122638706%7CUnknown%7CTWFpbGZsb3d8eyJWIjoiMC4wLjAwMDAiLCJQIjoiV2luMzIiLCJBTiI6Ik1haWwiLCJXVCI6Mn0%3D%7C1000&sdata=9N7DJZxaJYeM39MMx1Pe7Dns8sqboZycqEt7DZrqnRI%3D&reserved=0)). Please be aware that this license allows unrestricted use and distribution, even commercially, by third parties. Please reply and provide explicit written permission to publish XXX under a CC BY license and complete the attached form.”

Please upload the completed Content Permission Form or other proof of granted permissions as an "Other" file with your submission.

In the figure caption of the copyrighted figure, please include the following text: “Reprinted from [ref] under a CC BY license, with permission from [name of publisher], original copyright [original copyright year].”

b) If you are unable to obtain permission from the original copyright holder to publish these figures under the CC BY 4.0 license or if the copyright holder’s requirements are incompatible with the CC BY 4.0 license, please either i) remove the figure or ii) supply a replacement figure that complies with the CC BY 4.0 license. Please check copyright information on all replacement figures and update the figure caption with source information. If applicable, please specify in the figure caption text when a figure is similar but not identical to the original image and is therefore for illustrative purposes only.

The following resources for replacing copyrighted map figures may be helpful:

USGS National Map Viewer (public domain): [http://viewer.nationalmap.gov/viewer/](https://na01.safelinks.protection.outlook.com/?url=http%3A%2F%2Fviewer.nationalmap.gov%2Fviewer%2F&data=04%7C01%7C%7C2d969f51ff8740f942ad08d8c4903fb6%7C84df9e7fe9f640afb435aaaaaaaaaaaa%7C1%7C0%7C637475471122648700%7CUnknown%7CTWFpbGZsb3d8eyJWIjoiMC4wLjAwMDAiLCJQIjoiV2luMzIiLCJBTiI6Ik1haWwiLCJXVCI6Mn0%3D%7C1000&sdata=jfuZfBJpNfIKbNoY6991yiQ9Gvo33DMOIhRqd3vDsrs%3D&reserved=0)

The Gateway to Astronaut Photography of Earth (public domain): [http://eol.jsc.nasa.gov/sseop/clickmap/](https://na01.safelinks.protection.outlook.com/?url=http%3A%2F%2Feol.jsc.nasa.gov%2Fsseop%2Fclickmap%2F&data=04%7C01%7C%7C2d969f51ff8740f942ad08d8c4903fb6%7C84df9e7fe9f640afb435aaaaaaaaaaaa%7C1%7C0%7C637475471122648700%7CUnknown%7CTWFpbGZsb3d8eyJWIjoiMC4wLjAwMDAiLCJQIjoiV2luMzIiLCJBTiI6Ik1haWwiLCJXVCI6Mn0%3D%7C1000&sdata=uLRLO5e%2Bvq1o5o3JxzQRiRzcIyOyiCLkVq9mZ0lGAhc%3D&reserved=0)

Maps at the CIA (public domain): [https://www.cia.gov/library/publications/the-world-factbook/index.html](https://na01.safelinks.protection.outlook.com/?url=https%3A%2F%2Fwww.cia.gov%2Flibrary%2Fpublications%2Fthe-world-factbook%2Findex.html&data=04%7C01%7C%7C2d969f51ff8740f942ad08d8c4903fb6%7C84df9e7fe9f640afb435aaaaaaaaaaaa%7C1%7C0%7C637475471122658694%7CUnknown%7CTWFpbGZsb3d8eyJWIjoiMC4wLjAwMDAiLCJQIjoiV2luMzIiLCJBTiI6Ik1haWwiLCJXVCI6Mn0%3D%7C1000&sdata=yElb9bFTPA5daHjtmdZRbVE2ZjdWBNzcWghr1DV6KwA%3D&reserved=0) and [https://www.cia.gov/library/publications/cia-maps-publications/index.html](https://na01.safelinks.protection.outlook.com/?url=https%3A%2F%2Fwww.cia.gov%2Flibrary%2Fpublications%2Fcia-maps-publications%2Findex.html&data=04%7C01%7C%7C2d969f51ff8740f942ad08d8c4903fb6%7C84df9e7fe9f640afb435aaaaaaaaaaaa%7C1%7C0%7C637475471122658694%7CUnknown%7CTWFpbGZsb3d8eyJWIjoiMC4wLjAwMDAiLCJQIjoiV2luMzIiLCJBTiI6Ik1haWwiLCJXVCI6Mn0%3D%7C1000&sdata=IFNdOgNqycArDv9mQJ1Oq%2FHdImtAmQeXOfBQMwgpa%2FU%3D&reserved=0)

NASA Earth Observatory (public domain): [http://earthobservatory.nasa.gov/](https://na01.safelinks.protection.outlook.com/?url=http%3A%2F%2Fearthobservatory.nasa.gov%2F&data=04%7C01%7C%7C2d969f51ff8740f942ad08d8c4903fb6%7C84df9e7fe9f640afb435aaaaaaaaaaaa%7C1%7C0%7C637475471122668691%7CUnknown%7CTWFpbGZsb3d8eyJWIjoiMC4wLjAwMDAiLCJQIjoiV2luMzIiLCJBTiI6Ik1haWwiLCJXVCI6Mn0%3D%7C1000&sdata=e%2FJIldmhRkj4Uzd1N2izPBHhOABz1sfMIs%2BPwSE8kbM%3D&reserved=0)

Landsat: [http://landsat.visibleearth.nasa.gov/](https://na01.safelinks.protection.outlook.com/?url=http%3A%2F%2Flandsat.visibleearth.nasa.gov%2F&data=04%7C01%7C%7C2d969f51ff8740f942ad08d8c4903fb6%7C84df9e7fe9f640afb435aaaaaaaaaaaa%7C1%7C0%7C637475471122668691%7CUnknown%7CTWFpbGZsb3d8eyJWIjoiMC4wLjAwMDAiLCJQIjoiV2luMzIiLCJBTiI6Ik1haWwiLCJXVCI6Mn0%3D%7C1000&sdata=oEYF%2F%2Bej71ynEfknksh1cNhX%2B4DpZBSExrDo6qpr0hA%3D&reserved=0)

USGS EROS (Earth Resources Observatory and Science (EROS) Center) (public domain): [http://eros.usgs.gov/#](https://na01.safelinks.protection.outlook.com/?url=http%3A%2F%2Feros.usgs.gov%2F&data=04%7C01%7C%7C2d969f51ff8740f942ad08d8c4903fb6%7C84df9e7fe9f640afb435aaaaaaaaaaaa%7C1%7C0%7C637475471122678686%7CUnknown%7CTWFpbGZsb3d8eyJWIjoiMC4wLjAwMDAiLCJQIjoiV2luMzIiLCJBTiI6Ik1haWwiLCJXVCI6Mn0%3D%7C1000&sdata=%2Fxv%2BtDw8sJDicmnXT5GlVlIaMSpuTMJCCKWYslzNjmQ%3D&reserved=0)

Natural Earth (public domain): [http://www.naturalearthdata.com/](https://na01.safelinks.protection.outlook.com/?url=http%3A%2F%2Fwww.naturalearthdata.com%2F&data=04%7C01%7C%7C2d969f51ff8740f942ad08d8c4903fb6%7C84df9e7fe9f640afb435aaaaaaaaaaaa%7C1%7C0%7C637475471122678686%7CUnknown%7CTWFpbGZsb3d8eyJWIjoiMC4wLjAwMDAiLCJQIjoiV2luMzIiLCJBTiI6Ik1haWwiLCJXVCI6Mn0%3D%7C1000&sdata=s6o09bXMYJa1MeGIjRozjkCAUgKU%2BtvMKTVDtyPv1RI%3D&reserved=0)

**Response:** Thank you for the observation and detailed suggestions. Nevertheless, we elaborated the maps ourselves from the estimates of our study. It should be noted that Figure 2A and 2B are now “Figure 2” and “Figure” 3 in the final manuscript, respectively.

[Note: HTML markup is below. Please do not edit.]

**Reviewers' comments:**

Reviewer's Responses to Questions

**Comments to the Author**

1. Is the manuscript technically sound, and do the data support the conclusions?

The manuscript must describe a technically sound piece of scientific research with data that supports the conclusions. Experiments must have been conducted rigorously, with appropriate controls, replication, and sample sizes. The conclusions must be drawn appropriately based on the data presented.

Reviewer #1: Yes

2. Has the statistical analysis been performed appropriately and rigorously?

Reviewer #1: Yes

3. Have the authors made all data underlying the findings in their manuscript fully available?

The [PLOS Data policy](https://na01.safelinks.protection.outlook.com/?url=http%3A%2F%2Fwww.plosone.org%2Fstatic%2Fpolicies.action%23sharing&data=04%7C01%7C%7C2d969f51ff8740f942ad08d8c4903fb6%7C84df9e7fe9f640afb435aaaaaaaaaaaa%7C1%7C0%7C637475471122688679%7CUnknown%7CTWFpbGZsb3d8eyJWIjoiMC4wLjAwMDAiLCJQIjoiV2luMzIiLCJBTiI6Ik1haWwiLCJXVCI6Mn0%3D%7C1000&sdata=BePEaGEojFr0q1rg9tjkcKzq6hFunjzdzvKYjO30x0M%3D&reserved=0) requires authors to make all data underlying the findings described in their manuscript fully available without restriction, with rare exception (please refer to the Data Availability Statement in the manuscript PDF file). The data should be provided as part of the manuscript or its supporting information, or deposited to a public repository. For example, in addition to summary statistics, the data points behind means, medians and variance measures should be available. If there are restrictions on publicly sharing data—e.g. participant privacy or use of data from a third party—those must be specified.

Reviewer #1: Yes

4. Is the manuscript presented in an intelligible fashion and written in standard English?

PLOS ONE does not copyedit accepted manuscripts, so the language in submitted articles must be clear, correct, and unambiguous. Any typographical or grammatical errors should be corrected at revision, so please note any specific errors here.

Reviewer #1: Yes

5. Review Comments to the Author

Please use the space provided to explain your answers to the questions above. You may also include additional comments for the author, including concerns about dual publication, research ethics, or publication ethics. (Please upload your review as an attachment if it exceeds 20,000 characters)

Reviewer #1:

The manuscript uses MICE methods to estimate the proportion of children vaccinated by age group with each antigen in the routine infant schedule across 5 surveys in Mexico.

Table 1: Normally Hepatitis B after the birth dose is included in pentavalent vaccine. You have it listed separately. Please explain what the 5th antigen in pentavalent is if not hepatitis B?

**Response:**

In 1999, The national immunization program has incorporated the pentavalent whole-cell vaccine, which protects against diphtheria, tetanus, pertussis, hepatitis B and Haemophilus influenzae type b. Then, in 2007 the pentavalent whole-cell vaccine was replaced by the acellular pentavalent (PVac), which no longer contains the hepatitis B component and incorporates the inactivated poliomyelitis virus, in such a way that the hepatitis B vaccine is included separately^[1]^ ^[2]^ ^[3]^

Table 3: The n and % for sex are identical for ENSA 2000 and 2006. Is this correct?

**Response:** Thank you for the observation, it was our mistake, we changed the main manuscript**.**

Introduction 2nd paragraph: Calling EPI "outstanding" and "remarkable." It would be better to offer specific examples to demonstrate/quantify what you are referring to.

**Response:** Thank you for the observation. We changed the main manuscript**.**

Methods: It is unclear if some children are known to be completely unvaccinated and don't have a card for that reason. How were this instances handled in the model?

**Response:**

The caretaker of the children without a vaccination card answered the reminder questions, their self-reported coverage data are presented in S2 Table. It should be noted that the 2000 survey did not record the information of the group that did not have a vaccination card, thus data for this group was not reported in the table.

The surveys do not record the reason why they do not have the vaccination record, however, in a verbal interview with the person in charge of the survey design, he indicated that it was because the mothers left the records in the children's daycare centers. Another reason that could explain this is the increase in social insecurity and therefore the distrust of the population.

We can see that not having a card does not necessarily mean that a child has not been vaccinated, however, the information that is retrieved has many limitations and that when comparing it with the group that did present a card, differences of up to 90 percentage points are identified. In this sense, we decided to handle this group as missing data to estimate its vaccination status through the model.

Results: It would help the reader to know the estimated coverage by age group for each vaccine and survey among children who did present a vaccine card. Additionally, if verbal report from non-card presenting children, this would also be helpful, perhaps in the supplementary materials.

**Response:**

Thanks for the recommendation, we estimate for both groups, with and without vaccination card. These results can be found at the end of the document, which we also add as supplementary material.

It seems that some birth cohorts would have been considered in more than one survey. Did you consider presenting findings by birth cohort over time?

**Response:**

We did not consider it because our population of interest was those under five years of age and the surveys were conducted every six years, except for the 2010 and 2012 surveys.

Overall, there are several places where the language could be simplified or made clearer. One example is sentence 2, paragraph 1 of the introduction.

**Response:** Thanks for the recommendation, we did an additional review of English.

6. PLOS authors have the option to publish the peer review history of their article ([what does this mean?](https://na01.safelinks.protection.outlook.com/?url=https%3A%2F%2Fjournals.plos.org%2Fplosone%2Fs%2Feditorial-and-peer-review-process%23loc-peer-review-history&data=04%7C01%7C%7C2d969f51ff8740f942ad08d8c4903fb6%7C84df9e7fe9f640afb435aaaaaaaaaaaa%7C1%7C0%7C637475471122688679%7CUnknown%7CTWFpbGZsb3d8eyJWIjoiMC4wLjAwMDAiLCJQIjoiV2luMzIiLCJBTiI6Ik1haWwiLCJXVCI6Mn0%3D%7C1000&sdata=cQrfo3bUjJPLmC%2FYa5b4vVFyEGR5RGbn%2BClc%2FE%2B5qno%3D&reserved=0)). If published, this will include your full peer review and any attached files.


If you choose “no”, your identity will remain anonymous but your review may still be made public.


**Do you want your identity to be public for this peer review?** For information about this choice, including consent withdrawal, please see our [Privacy Policy](https://na01.safelinks.protection.outlook.com/?url=https%3A%2F%2Fwww.plos.org%2Fprivacy-policy&data=04%7C01%7C%7C2d969f51ff8740f942ad08d8c4903fb6%7C84df9e7fe9f640afb435aaaaaaaaaaaa%7C1%7C0%7C637475471122698670%7CUnknown%7CTWFpbGZsb3d8eyJWIjoiMC4wLjAwMDAiLCJQIjoiV2luMzIiLCJBTiI6Ik1haWwiLCJXVCI6Mn0%3D%7C1000&sdata=XnfHPGqcu14suD8fzyUCXLRRP9IuV61nuMl07CGywK0%3D&reserved=0).

Reviewer #1: No

[NOTE: If reviewer comments were submitted as an attachment file, they will be attached to this email and accessible via the submission site. Please log into your account, locate the manuscript record, and check for the action link "View Attachments". If this link does not appear, there are no attachment files.]

While revising your submission, please upload your figure files to the Preflight Analysis and Conversion Engine (PACE) digital diagnostic tool, [https://pacev2.apexcovantage.com/](https://na01.safelinks.protection.outlook.com/?url=https%3A%2F%2Fpacev2.apexcovantage.com%2F&data=04%7C01%7C%7C2d969f51ff8740f942ad08d8c4903fb6%7C84df9e7fe9f640afb435aaaaaaaaaaaa%7C1%7C0%7C637475471122708665%7CUnknown%7CTWFpbGZsb3d8eyJWIjoiMC4wLjAwMDAiLCJQIjoiV2luMzIiLCJBTiI6Ik1haWwiLCJXVCI6Mn0%3D%7C1000&sdata=b7VMI0n30rYtlPMGKavKQLTkYhHe6QiCsM5uB7y9tq4%3D&reserved=0). PACE helps ensure that figures meet PLOS requirements. To use PACE, you must first register as a user. Registration is free. Then, login and navigate to the UPLOAD tab, where you will find detailed instructions on how to use the tool. If you encounter any issues or have any questions when using PACE, please email PLOS at [figures@plos.org](mailto:figures@plos.org). Please note that Supporting Information files do not need this step.

*In compliance with data protection regulations, you may request that we remove your personal registration details at any time.* [*(Remove my information/details)*](https://na01.safelinks.protection.outlook.com/?url=https%3A%2F%2Fwww.editorialmanager.com%2Fpone%2Flogin.asp%3Fa%3Dr&data=04%7C01%7C%7C2d969f51ff8740f942ad08d8c4903fb6%7C84df9e7fe9f640afb435aaaaaaaaaaaa%7C1%7C0%7C637475471122708665%7CUnknown%7CTWFpbGZsb3d8eyJWIjoiMC4wLjAwMDAiLCJQIjoiV2luMzIiLCJBTiI6Ik1haWwiLCJXVCI6Mn0%3D%7C1000&sdata=ud4Xji8m7en3ZCj7sfIpkCk6txnN5UMG9NOHG1YleqA%3D&reserved=0)*. Please contact the publication office if you have any questions.*

1. Dagan R, Igbaria K, Piglansky L, Melamed R, Willems P, Grossi A, Kaufhold A. Safety and immunogenicity of a combined pentavalent diphtheria, tetanus, acellular pertussis, inactivated poliovirus and Haemophilus influenzae type b-tetanus conjugate vaccine in infants, compared with a whole cell pertussis pentavalent vaccine. Pediatr Infect Dis J. 1997 Dec;16(12):1113-21. doi: 10.1097/00006454-199712000-00004. PMID: 9427455.
2. Mexico introduces pentavalent vaccine. EPI Newsl. 1999 Aug;21(4):8. PMID: 12349263.
3. Pérez-Pérez GF, Rojas-Mendoza T, Cabrera-Gaytán DA, Grajales-Muñiz C. Pertussis in Mexico, an epidemiological overview. A study of 19 years at the Instituto Mexicano del Seguro Social. Rev Med Inst Mex Seg Soc. 2015 Mar-Apr;53(2):164-70.
